# A Study of Resistome in Mexican Chili Powder as a Public Health Risk Factor

**DOI:** 10.3390/antibiotics13020182

**Published:** 2024-02-13

**Authors:** Mayra Paola Mena Navarro, Merle Ariadna Espinosa Bernal, Claudia Alvarado Osuna, Miguel Ángel Ramos López, Aldo Amaro Reyes, Jackeline Lizzeta Arvizu Gómez, Juan Ramiro Pacheco Aguilar, Carlos Saldaña Gutiérrez, Victor Pérez Moreno, José Alberto Rodríguez Morales, María Carlota García Gutiérrez, Erika Álvarez Hidalgo, Jorge Nuñez Ramírez, José Luis Hernández Flores, Juan Campos Guillén

**Affiliations:** 1 Facultad de Química, Universidad Autónoma de Querétaro, Cerro de las Campanas S/N, Querétaro 76010, Mexico; mmena27@alumnos.uaq.mx (M.P.M.N.); mespinosa06@alumnos.uaq.mx (M.A.E.B.); miguel.angel.ramos@uaq.mx (M.Á.R.L.); aldo.amaro@uaq.edu.mx (A.A.R.); ramiro.pacheco@uaq.mx (J.R.P.A.); vperez@uaq.mx (V.P.M.); maria.carlota.garcia@uaq.edu.mx (M.C.G.G.); erika.beatriz.alvarez@uaq.mx (E.Á.H.); jorge.nunez@uaq.mx (J.N.R.); 2 Centro de Investigación y Asistencia en Tecnología y Diseño del Estado de Jalisco, Guadalajara 44270, Mexico; calvarado@ciatej.mx; 3 Secretaría de Investigación y Posgrado, Centro Nayarita de Innovación y Transferencia de Tecnología (CENITT), Universidad Autónoma de Nayarit, Tepic 63173, Mexico; jackeline.arvizu@uan.edu.mx; 4 Facultad de Ciencias Naturales, Universidad Autónoma de Querétaro, Av. De las Ciencias S/N, Querétaro 76220, Mexico; carlos.saldana@uaq.mx; 5 Facultad de Ingeniería, Universidad Autónoma de Querétaro, Cerro de las Campanas S/N, Querétaro 76010, Mexico; jose.alberto.rodriguez@uaq.mx; 6 Centro de Investigación y de Estudios Avanzados del IPN, Irapuato 36824, Mexico

**Keywords:** antibiotic resistance genes, microbiological quality, metagenomic, resistome profile, multidrug resistance, tetracycline, beta-lactam, aminoglycosides, *Bacillaceae*, *Enterobacteriaceae*

## Abstract

Chili powder is an important condiment around the world. However, according to various reports, the presence of pathogenic microorganisms could present a public health risk factor during its consumption. Therefore, microbiological quality assessment is required to understand key microbial functional traits, such as antibiotic resistance genes (ARGs). In this study, metagenomic next-generation sequencing (mNGS) and bioinformatics analysis were used to characterize the comprehensive profiles of the bacterial community and antibiotic resistance genes (ARGs) in 15 chili powder samples from different regions of Mexico. The initial bacterial load showed aerobic mesophilic bacteria (AMB) ranging between 6 × 10^3^ and 7 × 10^8^ CFU/g, sporulated mesophilic bacteria (SMB) from 4.3 × 10^3^ to 2 × 10^9^ CFU/g, and enterobacteria (En) from <100 to 2.3 × 10^6^ CFU/g. The most representative families in the samples were *Bacillaceae* and *Enterobacteriaceae*, in which 18 potential pathogen-associated species were detected. In total, the resistome profile in the chili powder contained 68 unique genes, which conferred antibiotic resistance distributed in 13 different classes. Among the main classes of antibiotic resistance genes with a high abundance in almost all the samples were those related to multidrug, tetracycline, beta-lactam, aminoglycoside, and phenicol resistance. Our findings reveal the utility of mNGS in elucidating microbiological quality in chili powder to reduce the public health risks and the spread of potential pathogens with antibiotic resistance mechanisms.

## 1. Introduction

Chili powder is a condiment used around the world and thus has high economic importance for key companies operating in the global market, with applications in food, beverages, cosmetics, personal care, and pharmaceuticals [1]. According to the FAO, the production of pulverized air-dried fruit of diverse varieties of chili pepper plants (*Capsicum* spp.) has seen significant growth in chili-producing countries such as India, China, Pakistan, and Bangladesh, which contribute about 55% of total world production [2]. In Mexico, chili powder production represents an important contribution to the North American market, and it is the most important spice in the Mexican diet; it is consumed directly, including in beverages or in various kinds of fast food, snacks, fruit cocktails, and almost all seasoned foods, regional sauces, and spicy candies [3,4,5].

However, according to several reports, microbiological safety is an important public health risk factor during chili powder consumption, which must be considered [6,7,8,9,10,11,12,13,14,15,16,17]. Therefore, it is necessary to implement a monitoring program to detect pathogenic microorganisms in chili powder that may cause food-borne illnesses in consumers or spread to other environments. Using microbial culture-dependent methods for bacterial community characterization has revealed that chili powder possesses a high diversity of microorganisms, including pathogenic species [18], a bacterial count of approximately 10^7^ CFU/g [6,7,8,9,10,11], and a spore-forming bacterial count in a range of 10^5^–10^7^ CFU/g [13,14]. Detections within this bacterial group include the *Bacillus subtilis* group [18] and the toxigenic *Bacillus cereus*, which has been detected in a low range (˂10^4^ cfu/g) but with significant relevance due to its toxigenic traits [17]. Also, pathogenic members of the *Enterobacteriaceae* family have been detected [19], one of which has historical relevance, having caused an outbreak of human salmonellosis in Germany associated with paprika-powdered potato chips [15].

Furthermore, concerning our research objective, it is of great significance that multidrug-resistant traits have been detected in bacteria associated with chili powder from diverse geographical regions [12,19]. Thus, for example, a previous study involving the analysis of antibiotic resistance profiles for opportunistic pathogens, such as *B. cereus s.l.* and *Kosakonia cowanii* isolated from Mexican chili powder, revealed their marked resistance to β-lactam antibiotics, trimethoprim–sulfamethoxazole, tetracycline, erythromycin, clindamycin, and chloramphenicol. Moreover, the presence of genes encoding extended-spectrum β-lactamases (ESBLs) and metallo-β-lactamases (MBLs) was also detected [19]. Therefore, the emergence of antibiotic resistance reservoirs and genes associated with the bacterial diversity in chili powder presents a very important health challenge and a significant public health concern. Studies such as those mentioned above are necessary to determine measures to avoid the spread of antibiotic resistance among the bacterial populations present through the natural mechanism of horizontal gene transfer, where plasmids, transposons, and integrons are important determinants in microorganisms that normally co-exist in the environment to acquire new resistance factors from different species [20].

Unfortunately, knowledge about the reservoirs of genes associated with antibiotic resistance in the bacterial populations present in chili powder is limited and needs detailed investigation. Therefore, the aim of the present study was to use shotgun metagenomic and bioinformatics analysis to understand the bacterial structure community profile and the associated resistome present in chili powder samples from some Mexican regions. We found mainly *Bacillaceae* and *Enterobacteriaceae* taxonomic groups with a higher abundance of multidrug-resistant genes associated with pathogenic species in chili powder. Our results will provide a better understanding of the resistome associated with the bacterial community structure in Mexican chili powder for the prevention and control of opportunistic pathogens.

## 2. Results

### 2.1. Bacterial Load

To understand the bacterial load in 15 chili powder samples collected during 2022 from six different geographical regions, we examined the results of the analysis of three bacterial groups. The content of aerobic mesophilic bacteria (AMB), sporulated mesophilic bacteria (SMB), and *Enterobacteriaceae* (En) are shown in Table 1. The AMB counts for the chili powder samples ranged from 6 × 10^3^ to 7 × 10^8^ CFU/g, and, based on the microbiological criteria for The International Commission on Microbiological Specifications for Foods (ICMSF) [21], six chili powder samples (CH2, CH3, CH5, CH7, CH8, and CH15) had marginal quality (10^4^–10^6^ CFU/g), while eight chili powder samples (CH1, CH4, CH6, CH9, CH10, CH11, CH12, and CH13) had values above 10^6^ CFU/g, which indicates unacceptable quality, and only one chili powder sample (CH14) had a value of ≤10^4^ CFU/g, which indicates acceptable quality. The results for the SMB counts ranged from 4.3 × 10^3^ to 2 × 10^9^ CFU/g. Although this bacterial group is not considered in the microbiological quality criteria for spices, the monitoring for *B. cereus* as a member of this group must be considered for its toxicity traits. An important bacterial group that serves as an indicator of hygienic quality is the *Enterobacteriaceae* family, and, according to the results, five chili powder samples (CH1, CH2, CH3, CH4, and CH7) exceeded the microbiological criteria for ICMSF (≤10^4^ CFU/g), while ten chili powder samples (CH5, CH6, CH8, CH9, CH10, CH11, CH12, CH13, CH14, and CH15) with values of ≤10^4^ CFU/g showed acceptable quality based on the ICMSF criteria for this group of microorganisms (Table 1). Therefore, based on the bacterial load results, we determined the bacterial diversity associated with these chili powder samples by using a culture-dependent approach and shotgun metagenome sequencing.

### 2.2. Bacterial Community Analysis

**Figure 1:**
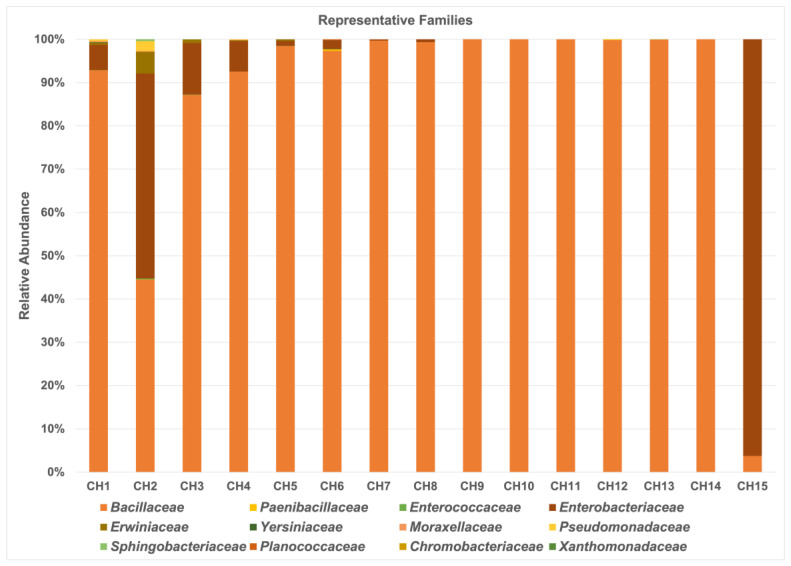
Taxonomic composition in fifteen chili powder samples. The bar graph represents the relative abundance of representative bacterial families as a percentage, with values being indicated by different colors.

The bacterial family taxa level distribution was represented by twelve groups but mainly by the *Bacillaceae* family, with a relative abundance average from 87.2% to 100% in 86.66% of chili powder samples (Figure 1). Only two chili powder samples (CH2 and CH15) showed lower relative abundance for the *Bacillaceae* family, with 44.5% and 3.77%, respectively. In addition, the *Enterobacteriaceae* family was represented in almost 60% of chili powder samples, with great significance and a high relative abundance; the samples CH2 and CH15 showed 47.34% and 96.23%, respectively, followed by CH3 with 11.86%, CH4 with 7.04%, and CH1 with 5.82%; and the other samples (CH5 to CH8) showed ˂2% of relative abundance. Other bacterial families represented in the samples, such as CH1, with a relative abundance lower than 0.5%, were *Erwiniaceae*, *Pseudomonadaceae*, *Moraxellaceae*, *Yersiniaceae*, and *Enterococcaceae*. In CH2, the *Erwiniaceae* family was represented by a relative abundance of 5.04%, followed by *Pseudomonadaceae* with 2.31%, while families such as *Sphingobacteriaceae*, *Moraxellaceae*, *Enterococcaceae*, *Xanthomonadaceae*, and *Paenibacillaceae* were lower than 0.5%. The *Erwiniaceae* family was also present in CH3, CH4, and CH5, with a relative abundance of 0.84%, 0.17%, and 0.37%, respectively. The *Planococcaceae* family was present only in CH6, with a relative abundance of 0.13%, and the *Chromobacteriaceae* family was present only in CH12, with 0.02%. Other chili powder samples that were present in the *Paenibacillaceae* family were CH6, CH12, and CH13, with a relative abundance lower than 0.4%.

**Figure 2:**
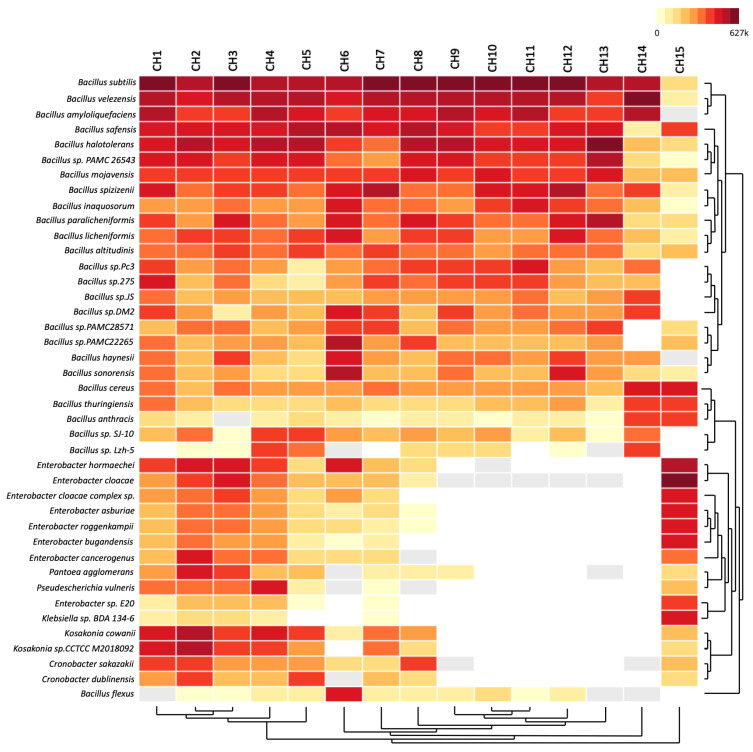
Heatmap of bacterial taxonomic composition in chili powder samples. Significant bacterial species composition was determined from the unique reads per million (rPM) that were mapped to specific microbial taxa using CZ-ID platform indicated by a colored logarithmic scale, with darker red representing the highest rPM.

**Figure 3:**
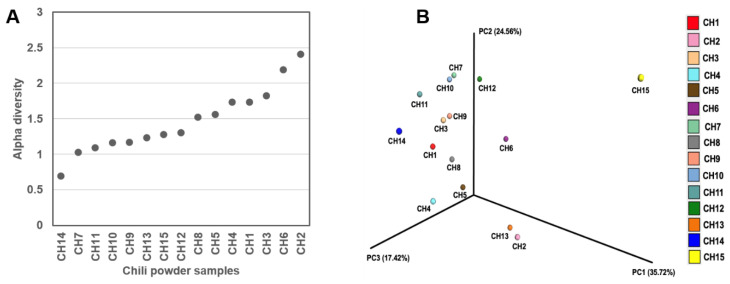
Alpha and beta diversity plots to visualize the difference of bacterial composition in chili powder samples. (**A**) Alpha diversity comparisons based on Shannon diversity index and (**B**) PCoA plot of beta diversity measure with the Bray–Curtis dissimilarity.

The bacterial genera detected in the chili powder samples are shown in Figure 2. The most commonly detected bacterial species belonged to the *Bacillaceae* family in almost all of the chili powder samples and were as follows: *B. subtilis*, *B. velezensis*, *B. amyloliquefaciens*, *B. safensis*, *B. halotolerans*, *B. mojavensis*, *B. spizizenii*, *B. inaquosorum*, *B. paralicheniformis*, *B. licheniformis*, *B. altitudinis*, *B. haynesii*, *B. sonorensis*, and some members of the *B. cereus* group. Regarding the chili powder samples that presented sequence hits for the *Enterobacteriaceae* family, the bacterial species were represented by *Enterobacter hormaechei*, *E. cloacae*, *E. asburiae*, *E. roggenkampii*, *E. bugandensis*, *E. cancerogenus*, *Pseudoscherichia vulneris*, *K. cowanii*, *Klebsiella* sp., *Cronobacter sakazakii*, and *C. dublinensis*, while the *Erwiniaceae* family was represented by *Pantoea agglomerans*. Alpha diversity analysis (Shannon diversity index) was performed to analyze the distribution of the bacterial community diversity present in all the chili powder samples; the results were plotted from a low to a high Shannon diversity index (Figure 3A), where the CH14 sample was the lower index and the CH2 sample was the higher index. Principal coordinates analysis (PCoA) based on Bray–Curtis dissimilarities was performed to express the differences between the bacterial communities from the chili powder samples (Figure 3B).

**Figure 4:**
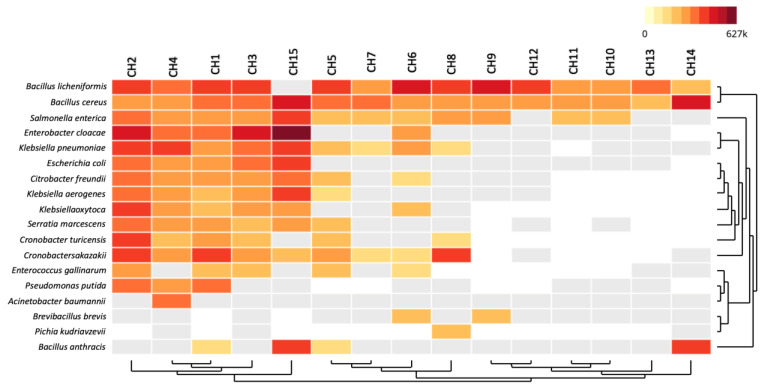
Heatmap of pathogen-related bacteria distribution in chili powder samples. Significant bacterial taxa composition was determined from the unique reads per million (rPM) using CZ-ID platform indicated by a colored logarithmic scale, with darker red representing the highest rPM.

According to the pathogen-related bacteria distribution in the chili powder samples using the CZ-ID platform, three principal sample groups were observed (Figure 4). The first group comprised the CH1, CH2, CH3, CH4, and CH15 samples, for which the read sequences (rPM) showed hits with pathogen-related species such as *B. licheniformis*, *B. cereus*, *S. enterica*, *E. cloacae*, *K. pneumoniae*, *E. coli*, *C. freundii*, *K. aerogenes*, *K. oxitoca*, *S. marcescens*, *C. turicensis*, *C. sakazakii*, *E. gallinarum*, *P. putida*, *A. baumannii*, and *B. anthracis*. The second group comprised CH5, CH6, CH7, and CH8, which presented pathogen-related bacteria such as *B. licheniformis*, *B. cereus*, *S. enterica*, *E. cloacae*, *K. pneumoniae*, *C. freundii*, *K. aerogenes*, *K. oxitoca*, *S. marcescens*, *C. turicensis*, *C. sakazakii*, *E. gallinarum*, *B. brevis*, *P. kudriavzevii*, and *B. anthracis*. The third group comprised CH9, CH10, CH11, CH12, CH13, and CH14, and the pathogen-related bacteria were principally *B. licheniformis*, *B. cereus*, *S. enterica*, and *B. anthracis*.

### 2.3. Resistome of Chili Powder

**Figure 5:**
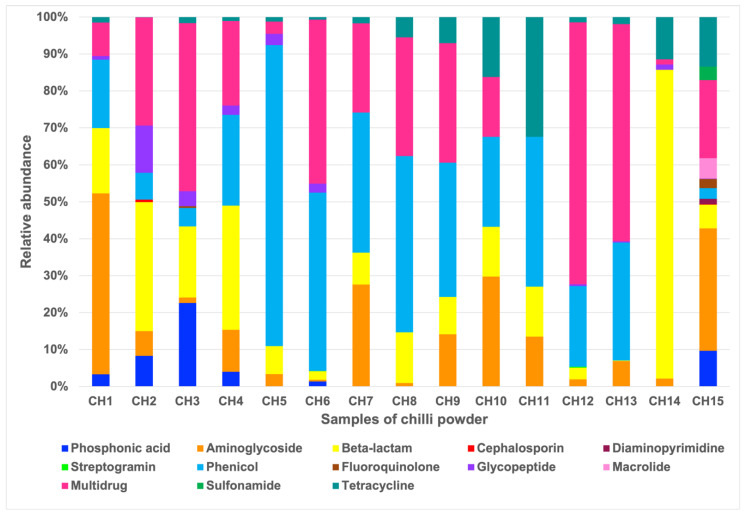
Relative abundance of antibiotic resistance classes. In total, 13 antibiotic resistance classes are indicated by graphic in 15 chili powder samples.

**Figure 6:**
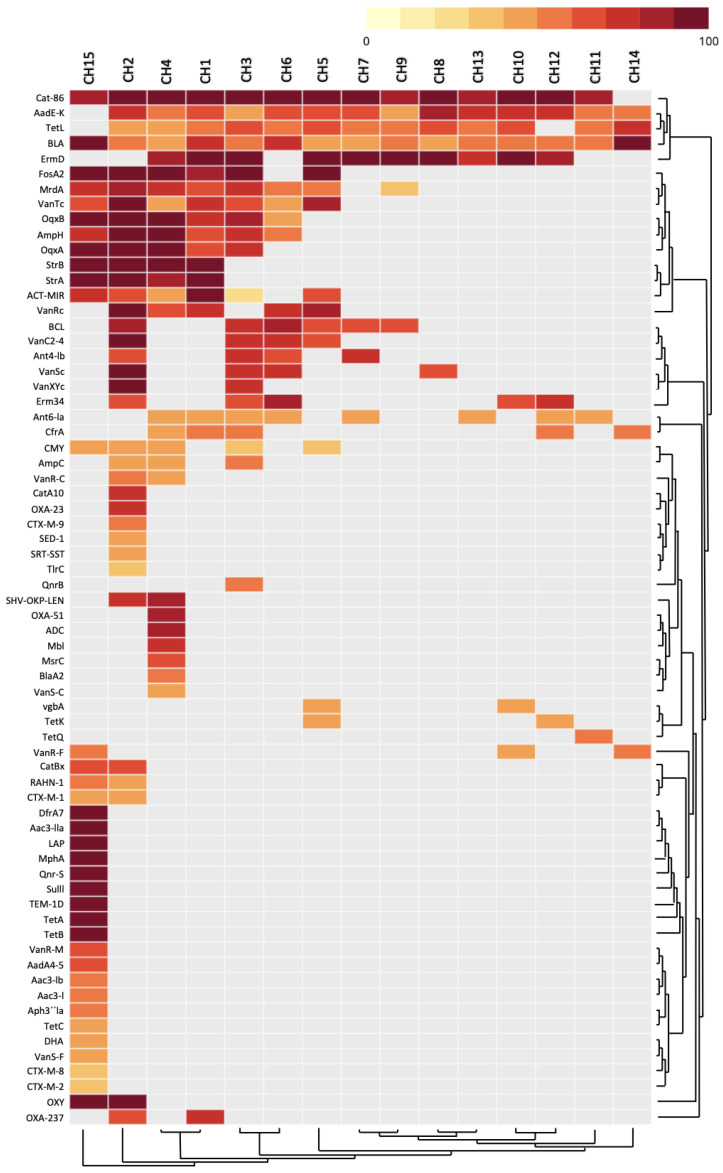
Heatmap of ARG distribution between chili powder samples. Results were obtained in CZ-ID platform and indicated by a colored scale, with darker red representing the highest readings.

According to the bacterial diversity structure present in the chili powder, we aimed to determine the resistome profiles, so an analysis of the prevalence of antibiotic resistance genes (ARGs) was reconstructed. In total, the resistome profiles of the chili powder contained 68 unique genes, which conferred resistance to antibiotics over 13 different classes (Figure 5 and Figure 6). Among the principal antibiotic resistance classes distributed with a high percentage of their presence in almost all the samples were multidrug, tetracycline, beta-lactam, aminoglycoside, and phenicol. The chili powder samples with a high relative abundance of resistance classes were CH15 with 12 classes, where aminoglycoside (33.14%), multidrug (21.10%), tetracycline (13.45%), and phosphonic acid (9.64%) showed a high relative abundance, while CH2 presented 8 resistance classes, where multidrug (29.26%), beta-lactam (34.91%), glycopeptide (12.79%), phenicol (7.29%), phosphonic acid (8.27%), and aminoglycoside (6.69%) showed a high relative abundance. Other chili powder samples, such as CH3, also presented eight resistance classes, where multidrug (45.47%), phosphonic acid (22.61%), and beta-lactam (19.28%) were the most abundant. CH6 presented eight resistance classes, where phenicol (48.29%) and multidrug (44.40%) were the most abundant. The remaining chili powder samples presented between four and seven resistance classes, where the high relative abundance of resistance classes was distributed across the multidrug, phenicol, beta-lactam, aminoglycoside, and tetracycline classes (Figure 5).

Figure 6 shows the ARG distribution between the chili powder samples. Among the ARGs represented in the phenicol resistance class and associated with bacteria genera, such as *Bacillus* and *Escherichia*, is chloramphenicol acetyltransferase (*cat86*, *catA10*, *catBx*), an enzyme that catalyzes the acetyl-CoA-dependent acetylation of chloramphenicol, causing inactivation. The aminoglycoside resistance class associated with bacteria genera, such as *Bacillus*, *Streptococcus*, *Staphylococcus*, *Enterococcus*, *Pseudomonas*, *Escherichia*, *Klebsiella*, *Salmonella*, *Erwinia* and *Xanthomonas*, is represented by aminoglycoside-modifying enzymes such as aminoglycoside nucleotidyltransferases (*aadA4-5*, *aadE-K*, *ant4-Ib*, *ant6-Ia*), aminoglycoside N-acetyltransferases (*aac3-I*, *aac3-Ib*, *aac3-IIa*) and aminoglycoside phosphotransferase (*aph3”Ia*, *strA*, *strB*). The energy-dependent efflux pump system of resistance to the tetracycline class associated with bacteria genera such as *Bacillus*, *Streptococcus*, *Staphylococcus*, *Enterococcus*, *Salmonella*, *Citrobacter*, *Escherichia*, and *Acinetobacter* is represented by *tetA*, *tetB*, *tetC*, *tetK*, *tetL*, and *tetQ*. The macrolide resistance class associated with bacteria genera such as *Acinetobacter*, *Bacillus*, *Citrobacter*, *Cronobacter*, *Enterobacter*, *Enterococccus*, *Escherichia*, *Klebsiella*, *Proteus*, *Pseudomonas*, *Streptococcus*, *Staphylococcus*, *Salmonella*, and *Serratia* is represented by the resistance-determinant genes *ermD* and *erm34*, which encode the rRNA methyltransferase, which methylates a specific residue on 23S rRNA, conferring macrolide–lincosamide–streptogramin B resistance. In addition, there are genes encoding macrolide efflux pumps, such as *msrC*, and macrolide inactivation, represented by the *mphA* gene encoding macrolide 2′-phosphotransferases and *tlrC*, which encode an F subfamily of ABC ATPase. The phosphonic acid resistance class is represented by the fosfomycin thiol transferase enzyme (*FosA2*) and associated with the *Enterobacter* genus. A multidrug efflux pump encoded by the *oqxA* and *oqxB* genes is also present and associated with bacteria genera such as *Citrobacter*, *Enterobacter*, *Escherichia*, *Klebsiella*, *Salmonella*, and *Shigella*, which confers resistance to multiple agents, such as olaquindox, mequindox, chloramphenicol, florfenicol, trimethoprim, ciprofloxacin, enrofloxacin, norfloxacin, tigecycline, and biocides. Resistance to glycopeptides, such as vancomycin, is represented by the *van* gene cluster (*vanC*, *vanR*, *vanS*, *vanT*, and *vanXY*) and associated with bacteria genera such as *Enterococcus* and *Klebsiella*, which produces resistance by altering the peptidoglycan target. Beta-lactam resistance is widely represented by genes encoding the beta-lactamase classes and is distributed in members of the *Enterobacteriaceae* and *Bacillaceae* families, such as CTX-M-1, CTX-M-2, CTX-M-8, *BLA*, *BLC*, *SHV-OKP-LEN*, Mbl, AmpC, AmpH, ACT-MIR, DHA, ADC LAP, OXY, TEM-1D, OXA-23, OXA-51, and OXA-237. There are also penicillin-binding proteins (PBPs), such as MrdA, in which sequence alterations have been related to beta-lactam resistance. The diaminopyrimidines resistance class is represented by DfrA7 and associated with *A. baumannii*, a dihydrofolate reductase enzyme critical for purine and thymidylate synthesis. The streptogramin resistance class is represented by VgbA (a lyase enzyme), and associated with *S. aureus*. CfrA is a 23S ribosomal RNA methyltransferase associated with bacteria genera such as *Enterococcus*, *Klebsiella*, and *Staphylococcus*, which confers resistance to some classes of antibiotics, including streptogramins, chloramphenicols, florfenicols, linezolids, and clindamycin. The fluoroquinolone resistance class is associated with members of the *Enterobacteriaceae* family such as *Escherichia* and *Klebsiella*, and is represented by the pentapeptide repeat proteins (QnrB and Qnr-S) that reduce susceptibility to quinolones by protecting the complex of DNA–DNA gyrase or DNA–topoisomerase IV enzymes. The sulfonamide resistance class mainly occurs through the acquisition of alternative genes encoding dihydropteroate synthase (DHPS) involved in nucleotide biosynthesis and is represented by SulII and associated with *Acinetobacter* genus in our results.

## 3. Discussion

The results of this study clearly show that, based on bacterial load analysis, the chili powder obtained from different geographical regions could have limited microbiological quality according to the ICMSF’s criteria, where values of ≤10^4^ CFU/g indicate acceptable quality. Therefore, our additional analysis results, using bacterial structure community profiles and the associated resistome present in the chili powder through shotgun metagenomic and bioinformatics analysis, provide, for the first time, a useful and potential additional test to better assess the quality of chili powder for chili-producing countries to give reliable validation of microbial public health risks.

According to the results, the PCA analyses and Shannon diversity index showed differences in the bacterial community composition in the chili powder, probably because the chili powder samples were collected from different geographical regions, and from a specific variety of plant of *Capsicum* spp. within each geographical region. Thus, the *Bacillaceae* family is one of the principal taxonomic groups that are present in 86.66% of the chili powder samples, with a high relative abundance (Figure 1 and Figure 2). Interestingly, several beneficial bacterial species were present in this bacterial group, such as the *B. subtilis* species complex (*B. subtilis*, *B. licheniformis*, *B. pumilus*, and *B. amyloliquefaciens*), which has been recognized as having diverse ecological functions, and some of whose members have great importance as plant growth promoters, biocontrol agents, probiotic and bioremediation agents, and in industry for their ability to produce important enzymes and antibiotics [22,23,24,25]. Also, in the operational group *B. amyloliquefaciens*, which comprises bacterial species detected in chili powder samples, there were the soil-borne *B. amyloliquefaciens* and the plant-associated *Bacillus velezensis* [24]. Some members of the *B. pumilus* group detected were *B. safensis* and *B. altitudinis*. Other species detected were *B. mojavensis* and *B. sonorensis*, which are close relatives of the *B. subtilis* clade [24]. Therefore, these findings reveal that chili powder possesses a high bacterial load of beneficial species with potential uses in biotechnology, which remain to be explored.

On the other hand, in the *Bacillaceae* family and as a potential health risk factor, two pathogen-related bacterial species, *B. licheniformis* and *B. cereus*, were detected in almost all of the chili powder samples (Figure 4). In the case of *B. licheniformis*, various related strains have been reported as opportunistic human pathogens that cause serious infections such as bacteremia, peritonitis, food poisoning, and eye infections [26]. However, more studies are necessary to understand the bacterial load and infection ability of this pathogen-related bacterial species detected in the chili powder samples. In accordance with other reports, toxigenic *B. cereus* strains have been detected and isolated as part of the microbial diversity in different spices, including chili powder [13,14,17,19]; based on our results, all the chili powder samples presented sequence hits for this pathogen-related bacterial species (Figure 4). Although *B. cereus* quantification was not detected by the culture-dependent approach used in this work, its toxin production capabilities suggest the necessity of implementing a monitoring program, because food-borne outbreaks have been associated with a *B. cereus* load above 10^5^ cfu/g of foodstuff [27]. According to the additional pathogen-related bacterial distribution (Figure 4), 80% of the chili powder samples could present a public health concern. Usually, members of the *Enterobacteriaceae* group are considered by food manufacturers as hygiene indicators for their ability to spread in diverse ecological niches, for their host range, and for their high relevance to the presence of virulence and antibiotic resistance genes, indicating pathogenic potential in humans, animals, insects, and plants [28]. In accordance with the bacterial load, in particular with the *Enterobacteriaceae* group (Table 1), the metagenomic detection of pathogen-related members of the *Enterobacteriaceae* family in the chili powder samples represents an important finding. However, more infection ability tests are necessary to understand its potential risks for consumers. Moreover, the mechanisms used for the pathogenic microorganism to invade the host and cause injury is a very extensive progressive process in which diverse physiological factors are involved. Therefore, the application of metagenomics to understand the bacterial community and prevent potential health risks is a first step of great importance in order to understand its spread and propose the best ways to avoid it in chili powder. Considering that pathogenic bacteria can spread through chili powder, and according to the bacterial genera detected and in order to enhance their importance, we have to consider that *E. coli* is a commensal organism present in the microbiota in the intestinal tract of humans and animals. Various strains have acquired virulence mechanisms as colonizers causing diarrhea and other intestinal diseases [28]. *S. enterica* usually causes gastroenteritis and is related to the consumption of contaminated foodstuffs [29], and an important human salmonellosis outbreak in Germany was associated with paprika-powdered potato chips [15]. *E. cloacae* is a common pathogen that can cause pneumonia, urinary tract infections, and septicemia [30]. *Klebsiella* spp. are among the significant opportunistic pathogens that have become a public health concern because they are a prevalent colonizer in human and animal microbiomes. According to some reports, 0.3% of all Gram-negative infections are represented by this bacterial microorganism, causing urinary tract infections, cystitis, pneumonia, surgical wound infections, endocarditis, and septicemia [31,32]. *C. freundii* is a microorganism that spreads in various environments, such as water, soil, food, and parts of the intestinal microbiota of animals and humans. Several related species have the ability to cause healthcare-associated infections, some involving the urinary tract, liver, biliary tract, peritoneum, intestines, respiratory tract, endocardium, soft tissue, meninges, and bloodstream [33]. *S. marcescens*, an opportunistic microorganism with the ability to spread easily in the environment and cause outbreaks, is relevant because it is associated with infections including those of the respiratory tract, bloodstream, central nervous system, urinary tract, and endocarditis [34]. Another opportunistic pathogen-related bacterial species detected in chili powder was *Cronobacter* spp., which belongs to the family *Enterobacteriaceae* and causes infections such as septicemia, pneumonia, meningitis, and necrotizing enterocolitis [35]. *E. gallinarum* causes a small fraction of healthcare-associated infections; however, it is considered a low-virulence microorganism that is associated principally with bloodstream, urinary tract, and surgical wound infections [36]. *P. putida* has recently become a public health concern due to its capacity to spread antibiotic resistance mechanisms; although it is considered to have low pathogenicity, it can cause infections in immunocompromised patients [37]. *A. baumannii* is a healthcare-associated pathogen with a high multidrug resistance and virulence, which is considered a major public health concern because it can cause death through its infection mechanisms. Infections associated with this bacterial species include skin and soft tissues, wound infections, bacteremia, endocarditis, urinary tract infections, meningitis, and pneumonia [38].

In addition to the detection of pathogen-related bacterial species, the results show that chili powder can provide a huge reservoir of antibiotic resistance genes and can be a potential route of the environmental transmission of antibiotic-resistant bacteria. In total, 68 associated ARGs, which confer bacterial resistance to at least 13 different classes of antibiotics, were detected in the 15 chili powder samples analyzed (Figure 5 and Figure 6). Previous studies of chili powder from different geographical regions demonstrated through PCR testing and sequencing a high prevalence of extended-spectrum β-lactamases (ESBLs) and metallo-β-lactamases (MBLs) in members of the *Enterobacteriaceae* and *Bacillaceae* families [17,19]. In addition, in a previous work from our research group [19], *B. cereus* isolated from Mexican chili powder showed resistance to penicillin G, ampicillin, carbenicillin, cefalotin, cefotaxime, dicloxacillin, amoxicillin/clavulanic acid, and trimethoprim-sulfamethoxazole in 100% of the isolates, to tetracycline in 90%, to erythromycin in 77%, to clindamycin in 74%, and to chloramphenicol in 42%. On the other hand, *K. cowanii*, a member of the *Enterobacteriaceae* family isolated from Mexican chili powder [19], showed resistance to penicillin, ampicillin, carbenicillin, erythromycin, clindamycin, and vancomycin in 100% of the isolates. Accordingly, these significant results were a key component in expanding the analysis. According to the results of this work, beta-lactam resistance genes are widely represented in 100% of the chili powder samples by genes encoding diverse beta-lactamase classes associated with the *Enterobacteriaceae* and *Bacillaceae* families, which could enable the hydrolysis of extended-spectrum cephalosporins, carbapenems, and penicillin, where bacterial genera associated with this resistance, such as *Acinetobacter*, *Bacillus*, *Citrobacter*, *Cronobacter*, *Enterobacter*, *Enterococccus*, *Escherichia*, *Klebsiella*, *Proteus*, *Pseudomonas*, *Streptococcus*, *Staphylococcus*, *Salmonella* and *Serratia*, could be a public health concern [39]. Some of these, for instance, CTX-Ms, are the most common ESBLs distributed globally in members of the *Enterobacteriaceae* family [39]. Also, OXA-type β-lactamases, which confer carbapenem resistance, were detected and are related to several species of *Acinetobacter*, which could be of particular interest in terms of public health [38,39]. ESBLs are easily transferable in a broad range of bacterial hosts, since they are mainly encoded by plasmids and mobile genetic elements such as integrons, insertion sequences, and transposons, so that its dissemination via horizontal transfer under specific conditions could be relevant as a major contributor in bacterial communities [40].

Multidrug resistance mediated by efflux pumps is widely conserved in bacteria; we found that some of these were encoded by the *oqxA* and *oqxB* genes. According to various reports, these genes are mainly associated with plasmid-mediated quinolone resistance (PMQR), which is widespread among members of the *Enterobacteriaceae* family and confers resistance to multiple antimicrobial agents, such as quinolones and fluoroquinolones, which are used during the clinical treatment of pathogenic bacterial strains such as *Citrobacter*, *Enterobacter*, *Escherichia*, *Klebsiella*, *Salmonella*, and *Shigella* [41,42,43]. Another mechanism of antibiotic resistance that was detected was to tetracycline through *tet* genes, which mainly encode efflux pumps and are present in both Gram-positive and Gram-negative bacteria, are widespread through plasmids and transposons, and can be transmitted through diverse genetic systems and spread in bacterial genera such as *Acinetobacter*, *Bacillus*, *Citrobacter*, *Cronobacter*, *Enterobacter*, *Enterococccus*, *Escherichia*, *Klebsiella*, *Proteus*, *Pseudomonas*, *Streptococcus*, *Staphylococcus*, *Salmonella*, and *Serratia* [44,45]. Another important mechanism detected was diverse antibiotic resistance genes that encode enzymes with modifying activity (Figure 6). These enzymes carry out the transfer of functional groups such as the acetyl (*cat86*, *catA10*, *catBx*, *aac3-I*, *aac3-Ib*, *aac3-IIa*), nucleotidyl (*aadA4-5*, *aadE-K*, *ant4-Ib*, *ant6-Ia*), phosphoryl (*aph3”Ia*, *strA and strB*), or thiol groups (*FosA2*), which confers resistance to bacterial genera such as *Bacillus*, *Streptococcus*, *Staphylococcus*, *Enterococcus*, *Pseudomonas*, *Escherichia*, *Klebsiella*, *Salmonella*, *Erwinia*, and *Xanthomonas*, and a range of antibiotics, including aminoglycosides, macrolides, and chloramphenicol, among many others [46,47]. Resistance to glycopeptides by the *van* gene cluster (*vanC*, *vanR*, *vanS*, *vanT*, and *vanXY*), which encodes enzymes that modify peptidoglycan, has been detected in several *Enterococci* species (*E. faecium*, *E. faecalis*, *E. gallinarum*), and the dissemination of vancomycin resistance among Gram-positive species via horizontal gene transfer is common, due to the presence of genetic determinants [48]. All the results indicate that chili powder possesses a high bacterial diversity with an extended ARG reservoir, and, therefore, more research is necessary for a better understanding of chili powder quality and potential public health risk.

## 4. Materials and Methods

### 4.1. Bacterial Load

We analyzed fifteen samples of chili powder elaborated from six varieties of *Capsicum annuum* L. and one sample from *Capsicum chinense* obtained during 2022 from different suppliers in six different geographical regions of Mexico (Table 1). Chili powder elaborated from *C. annuum*/Guajillo was obtained from San Luis Potosi, Fresnillo, Zacatecas and Jalisco. Chili powder elaborated from *C. annuum*/Mirasol was obtained from Zacatecas. Chili powder elaborated from *C. annuum*/De Árbol was obtained from San Luis Potosi, Querétaro and Yucatán. Chili powder elaborated from *C. annuum*/Poblano was obtained from Fresnillo, Zacatecas and Aguascalientes. Chili powder elaborated from *C. annuum*/Jalapeño and *C. annuum*/Morita was obtained from Fresnillo, Zacatecas. Chili powder elaborated from *C. chinense* was obtained from Yucatán. For representative samples, five samples of 250 g of each chili powder from different packets were mixed in a sterile plastic bag for 10 min; then, 250 g of the chili powder was collected in sterile Erlenmeyer flasks and kept at 10 °C until analysis. The aerobic mesophilic bacteria (AMB), sporulated mesophilic bacteria (SMB), and *Enterobacteriaceae* (En) counts from all the samples were determined according to Mexican regulations (NOM-092, NOM-113, and NOM-111) and the guidelines from the FDA’s Bacteriological Analytical Manual [49].

For these analyses, 1 g of each sample was added to 90 mL of peptone as diluent in an Erlenmeyer flask and the samples were homogenized for 5 min. Then, decimal dilutions were obtained and 100 µL was spread on duplicate plates of an appropriate medium, such as McConkey agar (Difco Laboratories; Detroit, MI, USA) for the Enterobacteria and tryptic soy agar (TSA) (Difco Laboratories; Detroit, MI, USA) for the AMB and SMB. The plates were incubated for 24–48 h at 37 °C and the growth of colonies was recorded as CFU/g of chili powder.

### 4.2. DNA Extraction

Two methodologies for metagenomic DNA extraction were used: the first was direct from the chili powder sample, and the second involved using a culture-dependent approach, where bacterial colonies were obtained from the TSA medium (Difco Laboratories; Detroit, MI, USA). For metagenomic DNA purification, the ZymoBIOMICS^TM^ DNA Miniprep Kit (Zymo Research, Irvine, CA, USA) was used. The integrity of the DNA was observed by agarose gel electrophoresis at 1%. Of the two methodologies, only the culture-dependent approach delivered high-quality DNA, whereas for the direct methodology, low concentration or degraded DNA was observed.

### 4.3. Shotgun Metagenomic Sequencing

The metagenomic DNA from the samples that underwent the culture-dependent approach were processed and sequenced with the ZymoBIOMICS^®^ shotgun metagenome sequencing service (Zymo Research, Irvine, CA, USA). In brief, Illumina^®^ DNA Library Prep Kit (Illumina, San Diego, CA, USA) was used with Nextera^®^ adapters (Illumina, San Diego, CA). The library was sequenced on the platform NovaSeq^®^ (Illumina, San Diego, CA, USA).

### 4.4. Bioinformatics Analysis

For bioinformatics analysis, the raw sequence reads were analyzed to remove low-quality reads (quality cutoff of 20 and size lower than 70 bp) and adapter sequences with Trimmomatic-0.33 [50]. The generated read (fastq) files were uploaded onto the CZ-ID platform (https://czid.org/ accessed in September 2023), which performs microbial composition and antimicrobial resistance database pipelines to detect and quantify bacterial pathogens [51]. For each chili powder sample, significant microbial composition was determined from the unique reads per million (rPM) that mapped to specific microbial taxa with the threshold filters as follows: nucleotide (NT) Z-score ≥ 1 (calculated from the mass-normalized background model created), NT rPM ≥ 10 (minimum of at least 10 reads of a mapping per 1 million reads to specific taxa), non-redundant protein (NR) rPM ≥ 5 (protein reads per million mapping to specific taxa), and average NT alignment ≥ 50 base pairs (average nucleotide read alignment mapping to specific taxa). Additional pipelines at the Bacterial and Viral Bioinformatics Resource Center (BV-BRC) for microbial composition, antimicrobial resistance database, and virulence factor database identification were used [52]. The resulting taxonomy and abundance information were further analyzed to perform alpha and beta diversity analyses using QIIME. The α diversity was measured with the Shannon diversity index [53,54]. The β diversity was measured with the Bray–Curtis index [55]. The Bray–Curtis dissimilarities were used for a principal coordinates analysis (PCoA).

### 4.5. Availability of Data

The raw metagenomic data have been deposited in the NCBI SRA database with accession number PRJNA1062060, as part of the BioProject.

## 5. Conclusions

Mexican chili powder is a very important condiment and represents an important source of rising income, with many chili-producing geographical regions continuing to support demand. Thus, it is necessary to promote a safe environment that encourages the achievement and continuous improvement of chili powder quality, in particular in terms of microbiological criteria, for which pathogen detection must be a priority to avoid spread during consumption. The results obtained in this work demonstrate that chili powder has an important bacterial diversity with a huge reservoir of antibiotic resistance genes. Understanding these results will provide future research directions for decreasing the public health risks associated with pathogenic microorganisms, which present diverse antibiotic resistance mechanisms.

## Figures and Tables

**Table 1:** Bacterial load analysis of chili powder samples from different geographical regions in Mexico.

SAMPLE	Species/Variety	Geographic Region	AMB (CFU/g)	SMB (CFU/g)	En (CFU/g)
CH1	*C. annuum*/Guajillo	San Luis Potosí	2.3 × 10^7^	2.2 × 10^7^	1.1 × 10^6^
CH2	*C. annuum*/Mirasol	Zacatecas, Zac.	1.2 × 10^6^	3.5 × 10^5^	1.2 × 10^5^
CH3	*C. annuum*/Guajillo	Fresnillo, Zac.	6.1 × 10^6^	2.9 × 10^6^	5.1 × 10^5^
CH4	*C. annuum*/De Árbol	San Luis Potosí	2.1 × 10^7^	3 × 10^7^	2.3 × 10^6^
CH5	*C. annuum*/Mirasol	Zacatecas, Zac.	5.8 × 10^6^	3.2 × 10^7^	2.1 × 10^4^
CH6	*C. annuum*/Xkatik	Yucatán	1 × 10^7^	5.1 × 10^8^	˂10^2^
CH7	*C. annuum*/Guajillo	Jalisco	4.9 × 10^6^	4.7 × 10^7^	4.3 × 10^5^
CH8	*C. annuum*/Jalapeño	Fresnillo, Zac.	1.7 × 10^5^	1.9 × 10^6^	<10^2^
CH9	*C. annuum*/Poblano	Fresnillo, Zac.	1 × 10^7^	1.2 × 10^7^	<10^2^
CH10	*C. annuum*/Morita	Fresnillo, Zac.	2 × 10^7^	1.4 × 10^7^	<10^2^
CH11	*C. annuum*/Guajillo	San Luis Potosí	7 × 10^8^	1.2 × 10^7^	<10^2^
CH12	*C. annuum*/De Árbol	Yucatán	4.4 × 10^7^	2 × 10^9^	<10^2^
CH13	*C. chinense*	Yucatán	3.8 × 10^7^	3.3 × 10^7^	7.4 × 10^4^
CH14	*C. annuum*/Poblano	Aguascalientes	6 × 10^3^	1.7 × 10^4^	<10^2^
CH15	*C. annuum*/De Árbol	Querétaro	1 × 10^6^	4.3 × 10^3^	4.8 × 10^3^

## Data Availability

The data presented in this work are available from the corresponding authors upon request.

## References

[1] Baenas N., Belović M., Ilic N., Moreno D.A., García-Viguera C. (2019). Industrial use of pepper (*Capsicum annum* L.) derived products: Technological benefits and biological advantages. Food Chem..

[2] (2023). FAOSTAT. http://www.fao.org.

[3] Aguilar-Meléndez A., Vásquez-Dávila M.A., Manzanero-Medina G.I., Katz E. (2021). Chile (*Capsicum* spp.) as Food-Medicine Continuum in Multiethnic Mexico. Foods.

[4] Rincón V.H.A., Torres T.C., López P.L., Moreno L.L., Meraz M.R., Mendoza H.V., Castillo J.A.A. (2010). Los Chiles de México y su Distribución.

[5] García-Gaytán V., Gómez-Merino F.C., Trejo-Téllez L.I., Baca-Castillo G.A., García-Morales S. (2017). The Chilhuacle Chili (*Capsicum annuum* L.) in Mexico: Description of the Variety, Its Cultivation, and Uses. Int. J. Agron..

[6] Molnár H., Bata-Vidács I., Baka E., Cserhalmi Z., Ferenczi S., Tömösközi-Farkas R., Adányi N., Székács A. (2018). The effect of different decontamination methods on the microbial load, bioactive components, aroma and colour of spice paprika. Food Control.

[7] Feroz F., Shimizu H., Nishioka T., Mori M., Sakagami Y. (2016). Bacterial and Fungal Counts of Dried and Semi-Dried Foods Collected from Dhaka, Bangladesh, and Their Reduction Methods. Biocontrol Sci..

[8] Van Doren J.M., Neil K.P., Parish M., Gieraltowski L., Gould L.H., Gombas K.L. (2013). Foodborne illness outbreaks from microbial contaminants in spices, 1973–2010. Food Microbiol..

[9] González M.G.M., Romero S.M., Arjona M., Larumbe A.G., Vaamonde G. (2017). Microbiological quality of Argentinian paprika. Rev. Argent. Microbiol..

[10] Mamun A.A., Masuma A., Majumder D., Ali M., Hossen M., Maruf K. (2016). Quality assessment of selected commercial brand of chilli powder in Bangladesh. MOJ Food Process. Technol..

[11] Bata-Vidács I., Baka E., Tóth Á., Csernus O., Luzics S., Adányi N., Székács A., Kukolya J. (2018). Investigation of regional differences of the dominant microflora of spice paprika by molecular methods. Food Control.

[12] György É., Laslo É., Antal M., András C.D. (2021). Antibiotic resistance pattern of the allochthonous bacteria isolated from commercially available spices. Food Sci. Nutr..

[13] Frentzel H., Kraushaar B., Krause G., Bódi D., Wichmann-Schauer H., Appel B., Mader A. (2018). Phylogenetic and toxinogenic characteristics of *Bacillus cereus* group members isolated from spices and herbs. Food Control.

[14] Hariram U., Labbé R. (2015). Spore prevalence and toxigenicity of Bacillus cereus and Bacillus thuringiensis isolates from U.S. retail spices. J. Food Prot..

[15] Lehmacher A., Bockemühl J., Aleksic S. (1995). Nationwide outbreak of human salmonellosis in Germany due to contaminated paprika and paprika-powdered potato chips. Epidemiol. Infect..

[16] Banerjee M., Sarkar P.K. (2003). Microbiological quality of some retail spices in India. Food Res. Int..

[17] Hernández A.G.C., Ortiz V.G., Gómez J.L.A., López M.Á.R., Morales J.A.R., Macías A.F., Hidalgo E.Á., Ramírez J.N., Gallardo F.J.F., Gutiérrez M.C.G. (2021). Detection of *Bacillus cereus sensu lato* Isolates Posing Potential Health Risks in Mexican Chili Powder. Microorganisms.

[18] Moore R.E., Millar B.C., Panickar J.R., Moore J.E. (2019). Microbiological safety of spices and their interaction with antibiotics: Implication for antimicrobial resistance and their role as potential antibiotic adjuncts. Food Qual. Saf..

[19] Hernández Gómez Y.F., González Espinosa J., Ramos López M.Á., Arvizu Gómez J.L., Saldaña C., Rodríguez Morales J.A., García Gutiérrez M.C., Pérez Moreno V., Álvarez Hidalgo E., Nuñez Ramírez J. (2022). Insights into the Bacterial Diversity and Detection of Opportunistic Pathogens in Mexican Chili Powder. Microorganisms.

[20] Walsh F., Duffy B. (2013). The Culturable Soil Antibiotic Resistome: A Community of Multi-Drug Resistant Bacteria. PLoS ONE.

[21] ICSMF (International Commission on Microbiological Specifications for Foods) (1986). Microorganisms in Foods 2, Sampling Formicrobiological Analysis: Principles and Specific Applications.

[22] Caulier S., Nannan C., Gillis A., Licciardi F., Bragard C., Mahillon J. (2019). Overview of the antimicrobial compounds produced by members of the *Bacillus subtilis* group. Front. Microbiol..

[23] Su Y., Liu C., Fang H., Zhang D. (2020). *Bacillus subtilis*: A universal cell factory for industry, agriculture, biomaterials and medicine. Microb. Cell Factories.

[24] Iqbal S., Begum F., Rabaan A.A., Aljeldah M., Al Shammari B.R., Alawfi A., Alshengeti A., Sulaiman T., Khan A. (2023). Classification and Multifaceted Potential of Secondary Metabolites Produced by *Bacillus subtilis* Group: A Comprehensive Review. Molecules.

[25] Ngalimat M.S., Yahaya R.S.R., Baharudin M.M.A.-A., Yaminudin S.M., Karim M., Ahmad S.A., Sabri S. (2021). A Review on the Biotechnological Applications of the Operational Group *Bacillus amyloliquefaciens*. Microorganisms.

[26] Haydushka I.A., Markova N., Kirina V., Atanassova M. (2012). Recurrent sepsis due to *Bacillus licheniformis*. J. Glob. Infect. Dis..

[27] EFSA (2016). Panel on biological hazards (BIOHAZ). Scientific opinion on the risks for public health related to the presence of Bacillus cereus and other *Bacillus* spp. including *Bacillus thuringiensis* in foodstuffs. EFSA J..

[28] Janda J.M., Abbott S.L. (2021). The changing face of the family *Enterobacteriaceae* (order: “Enterobacterales”): New members, taxonomic issues, geographic expansion, and new diseases and disease syndromes. Clin. Microbiol. Rev..

[29] Alvarez D.M., Barrón-Montenegro R., Conejeros J., Rivera D., Undurraga E.A., Moreno-Switt A.I. (2023). A review of the global emergence of multidrug-resistant *Salmonella enterica* subsp. enterica Serovar Infantis. Int. J. Food Microbiol..

[30] Annavajhala M.K., Gomez-Simmonds A., Uhlemann A.C. (2019). Multidrug-resistant Enterobacter cloacae complex emerging as a global, diversifying threat. Front. Microbiol..

[31] Navon-Venezia S., Kondratyeva K., Carattoli A. (2017). *Klebsiella pneumoniae*: A major worldwide source and shuttle for antibiotic resistance. FEMS Microbiol. Rev..

[32] Effah C.Y., Sun T., Liu S., Wu Y. (2020). *Klebsiella pneumoniae*: An increasing threat to public health. Ann. Clin. Microbiol. Antimicrob..

[33] Liu L.H., Wang N.Y., Wu AY J., Lin C.C., Lee C.M., Liu C.P. (2018). *Citrobacter freundii* bacteremia: Risk factors of mortality and prevalence of resistance genes. J. Microbiol. Immunol. Infect..

[34] Fernández A.L., Adrio B., Cereijo J.M.M., Monzonis M.A.M., El-Diasty M.M., Escudero J.A. (2020). Clinical study of an outbreak of postoperative mediastinitis caused by *Serratia marcescens* in adult cardiac surgery. Interact. Cardiovasc. Thorac. Surg..

[35] Lu Y., Liu P., Li C., Sha M., Fang J., Gao J., Xu X., Matthews K.R. (2019). Prevalence and genetic diversity of *Cronobacter* species isolated from four infant formula production factories in China. Front. Microbiol..

[36] Monticelli J., Knezevich A., Luzzati R., Di Bella S. (2018). Clinical management of non-faecium non-faecalis vancomycin-resistant enterococci infection. Focus on *Enterococcus gallinarum* and *Enterococcus casseliflavus*/*flavescens*. J. Infect. Chemother..

[37] Tan G., Xi Y., Yuan P., Sun Z., Yang D. (2019). Risk factors and antimicrobial resistance profiles of *Pseudomonas putida* infection in Central China, 2010–2017. Medicine.

[38] Ibrahim S., Al-Saryi N., Al-Kadmy I.M., Aziz S.N. (2021). Multidrug-resistant *Acinetobacter baumannii* as an emerging concern in hospitals. Mol. Biol. Rep..

[39] Bush K., Bradford P.A. (2020). Epidemiology of β-lactamase-producing pathogens. Clin. Microbiol. Rev..

[40] De Angelis G., Del Giacomo P., Posteraro B., Sanguinetti M., Tumbarello M. (2020). Molecular Mechanisms, Epidemiology, and Clinical Importance of β-Lactam Resistance in *Enterobacteriaceae*. Int. J. Mol. Sci..

[41] Li J., Zhang H., Ning J., Sajid A., Cheng G., Yuan Z., Hao H. (2019). The nature and epidemiology of OqxAB, a multidrug efflux pump. Antimicrob. Resist. Infect. Control.

[42] Bharatham N., Bhowmik P., Aoki M., Okada U., Sharma S., Yamashita E., Shanbhag A.P., Rajagopal S., Thomas T., Sarma M. (2021). Structure and function relationship of OqxB efflux pump from *Klebsiella pneumoniae*. Nat. Commun..

[43] Moosavian M., Khoshkholgh Sima M., Ahmad Khosravi N., Abbasi Montazeri E. (2021). Detection of OqxAB Efflux Pumps, a Multidrug-Resistant Agent in Bacterial Infection in Patients Referring to Teaching Hospitals in Ahvaz, Southwest of Iran. Int. J. Microbiol..

[44] Nøhr-Meldgaard K., Struve C., Ingmer H., Agersø Y. (2021). The Tetracycline Resistance Gene, tet(W) in *Bifidobacterium animalis* subsp. lactis Follows Phylogeny and Differs From tet(W) in Other Species. Front. Microbiol..

[45] Grossman T.H. (2016). Tetracycline Antibiotics and Resistance. Cold Spring Harb. Perspect. Med..

[46] Schaenzer A.J., Wright G.D. (2020). Antibiotic Resistance by Enzymatic Modification of Antibiotic Targets. Trends Mol. Med..

[47] Varela M.F., Stephen J., Lekshmi M., Ojha M., Wenzel N., Sanford L.M., Hernandez A.J., Parvathi A., Kumar S.H. (2021). Bacterial Resistance to Antimicrobial Agents. Antibiotics.

[48] Stogios P.J., Savchenko A. (2020). Molecular mechanisms of vancomycin resistance. Protein Sci..

[49] Bacteriological Analytical Manual Online. Center for Food Safety and Applied Nutrition.

[50] Bolger A.M., Lohse M., Usadel B. (2014). Trimmomatic: A flexible trimmer for Illumina sequence data. Bioinformatics.

[51] Kalantar K.L., Carvalho T., De Bourcy C.F.A., Dimitrov B., Dingle G., Egger R., Han J., Holmes O.B., Juan Y.-F., King R. (2020). IDseq—An open source cloud-based pipeline and analysis service for metagenomic pathogen detection and monitoring. Gigascience.

[52] Olson R.D., Assaf R., Brettin T., Conrad N., Cucinell C., Davis J.J., Dempsey D.M., Dickerman A., Dietrich E.M., Kenyon R.W. (2022). Introducing the Bacterial and Viral Bioinformatics Resource Center (BV-BRC): A resource combining PATRIC, IRD and ViPR. Nucleic Acids Res..

[53] Chao A. (1984). Nonparametric Estimation of the Number of Classes in a Population. Scand. J. Stat..

[54] Shannon C.E. (1948). A Mathematical Theory of Communication. Bell Syst. Tech. J..

[55] Bray J.R., Curtis J.T. (1957). An Ordination of the Upland Forest Communities of Southern Wisconsin. Ecol. Monogr..

